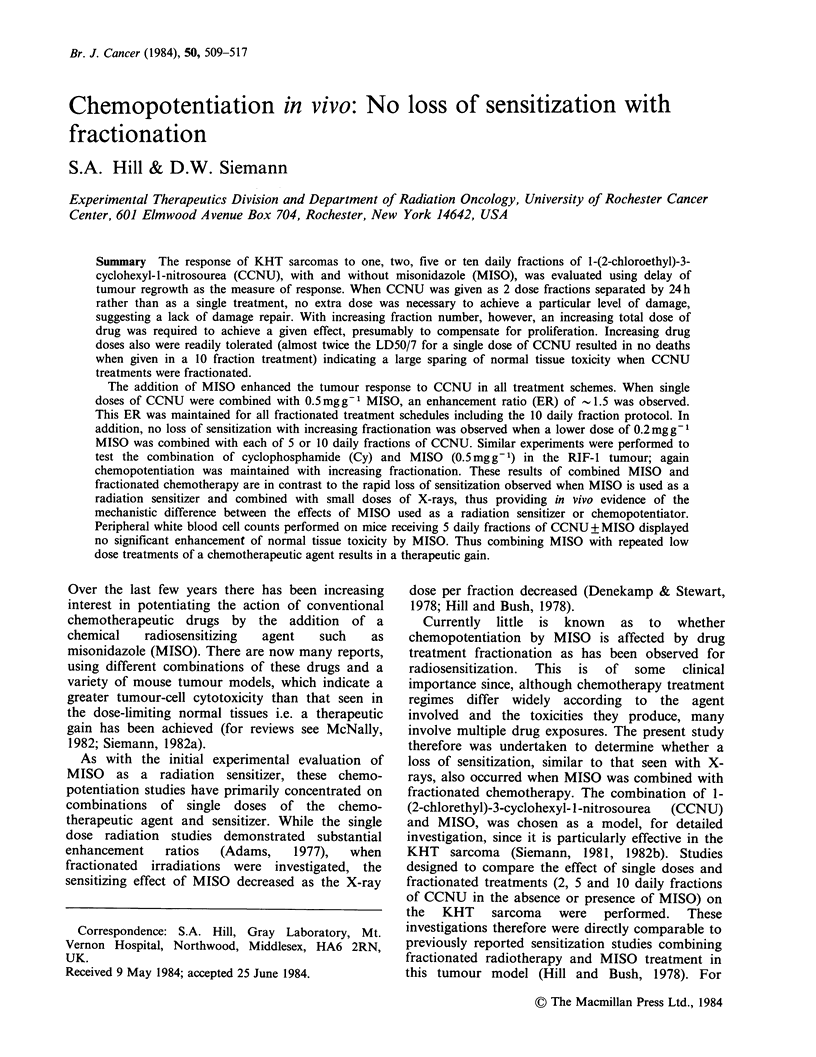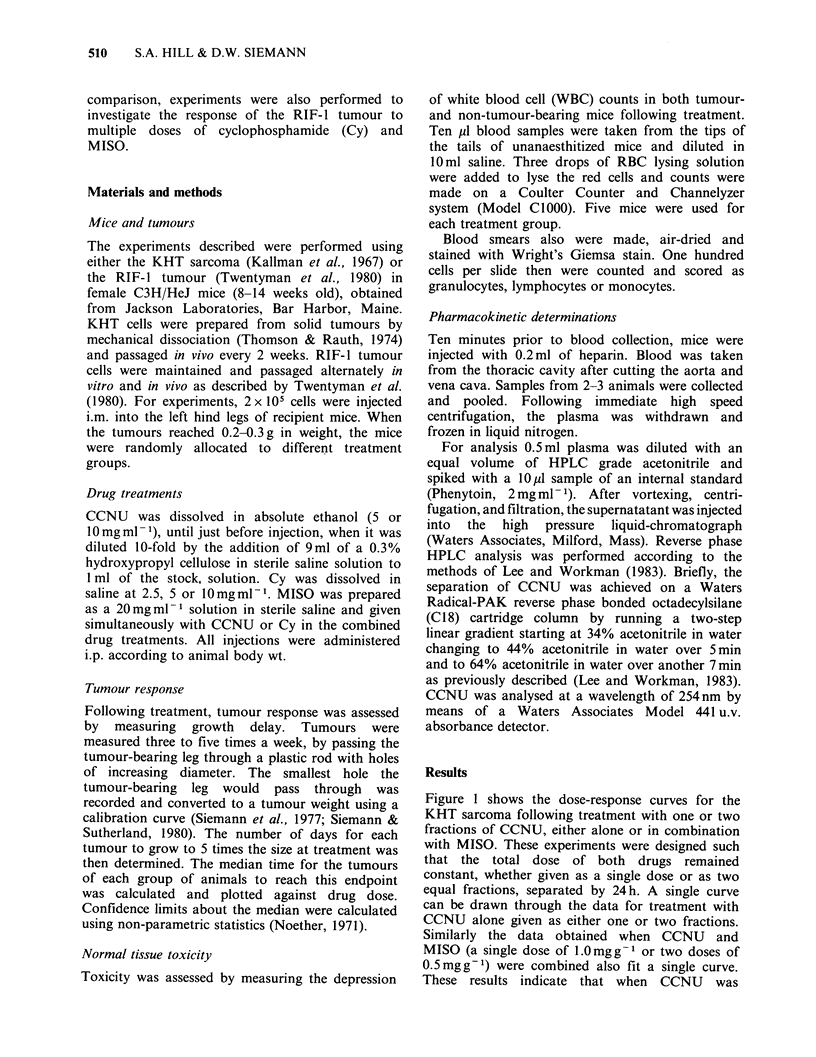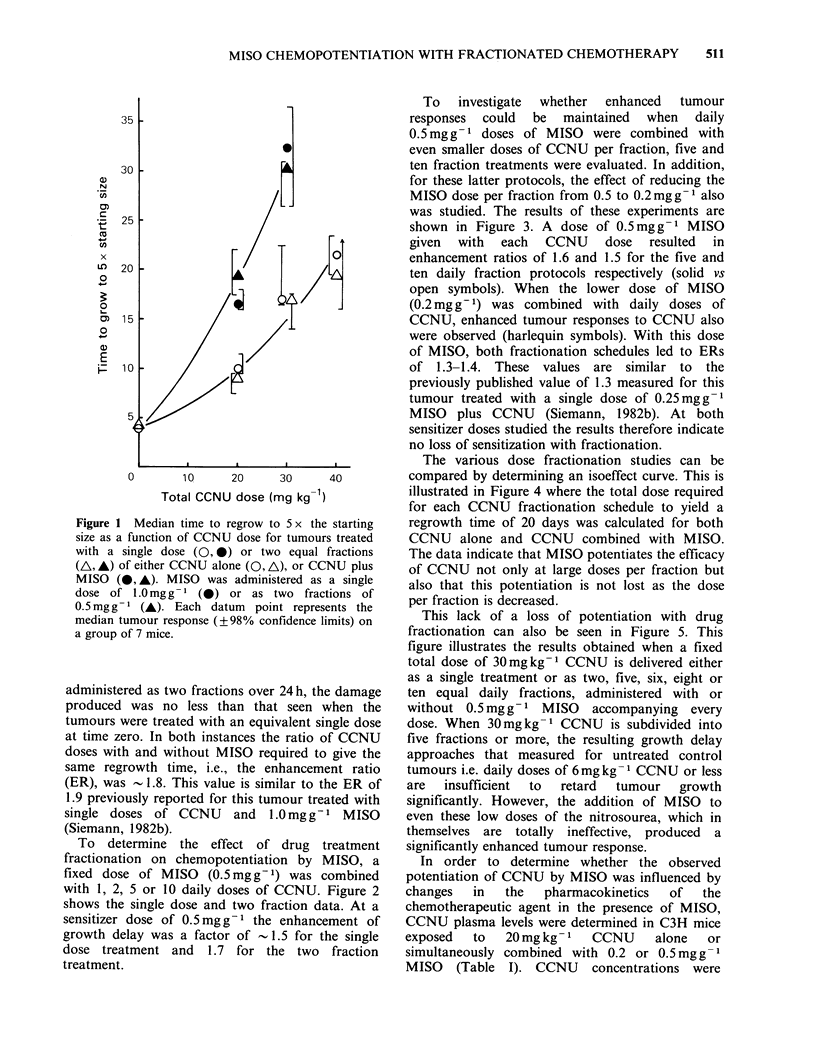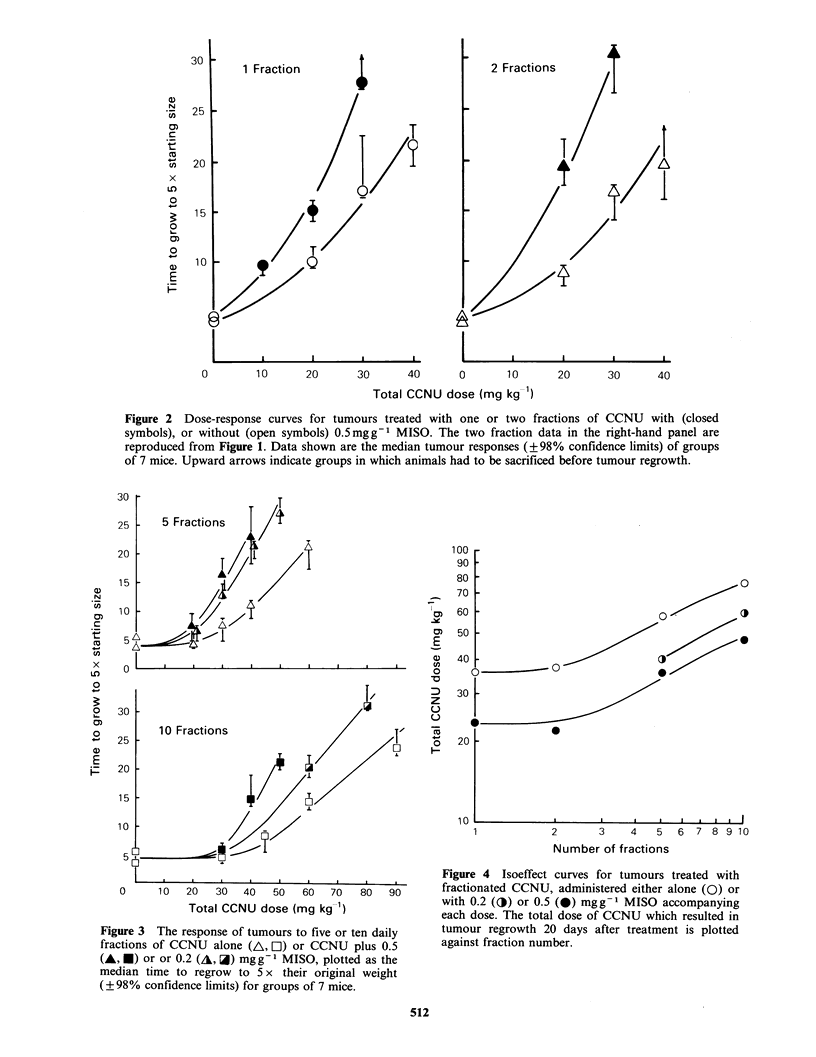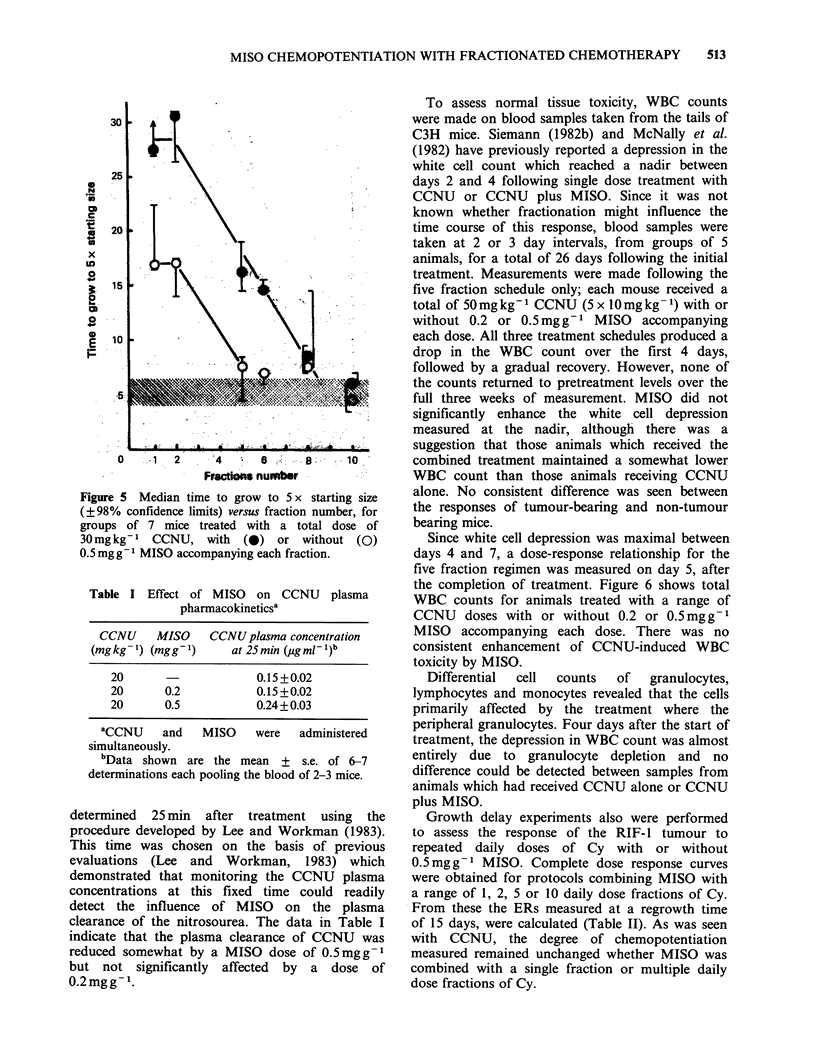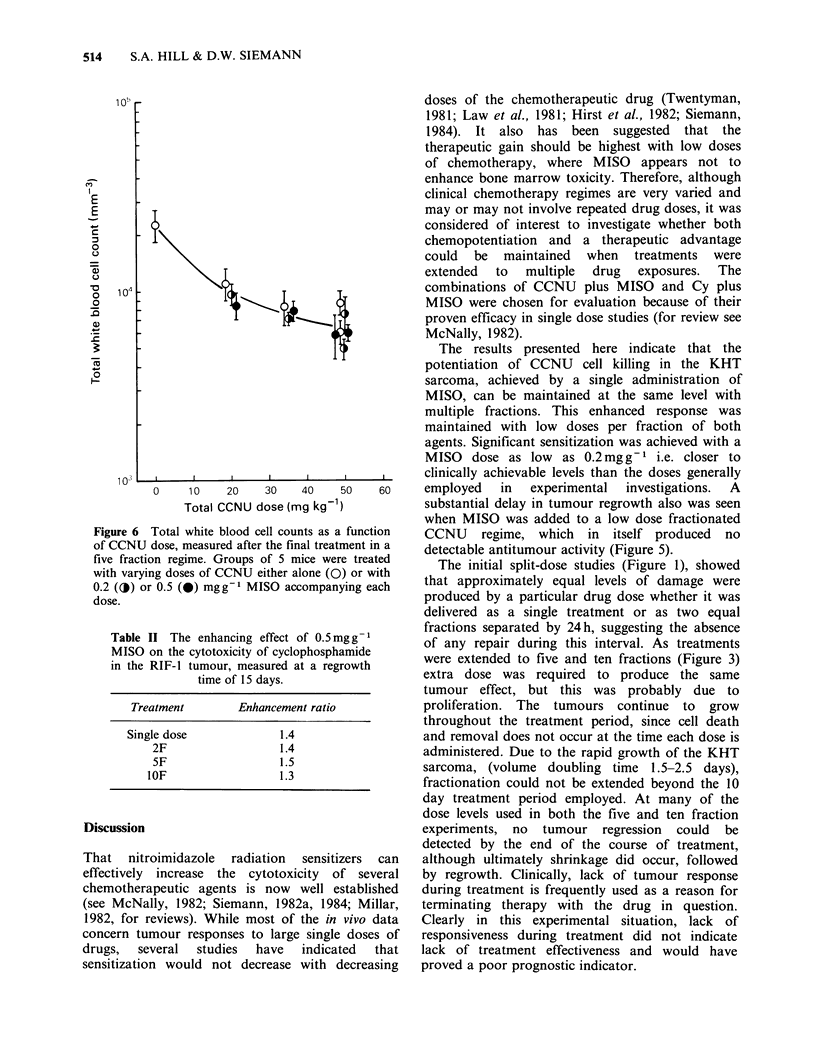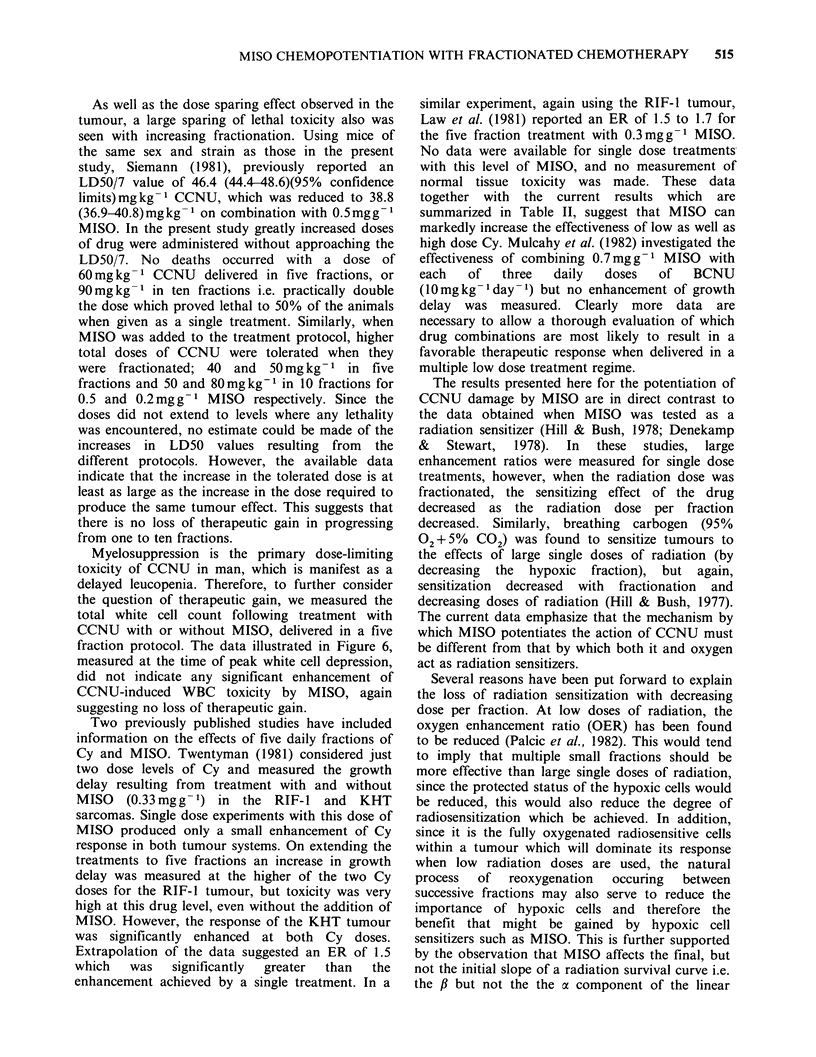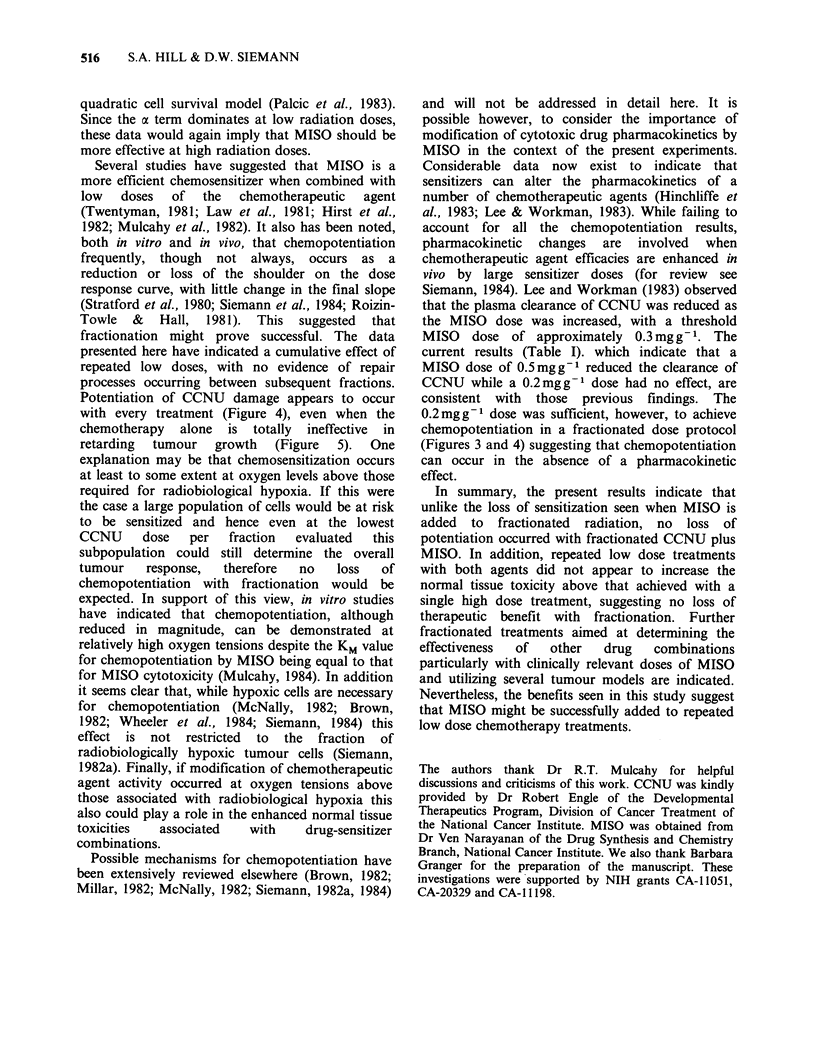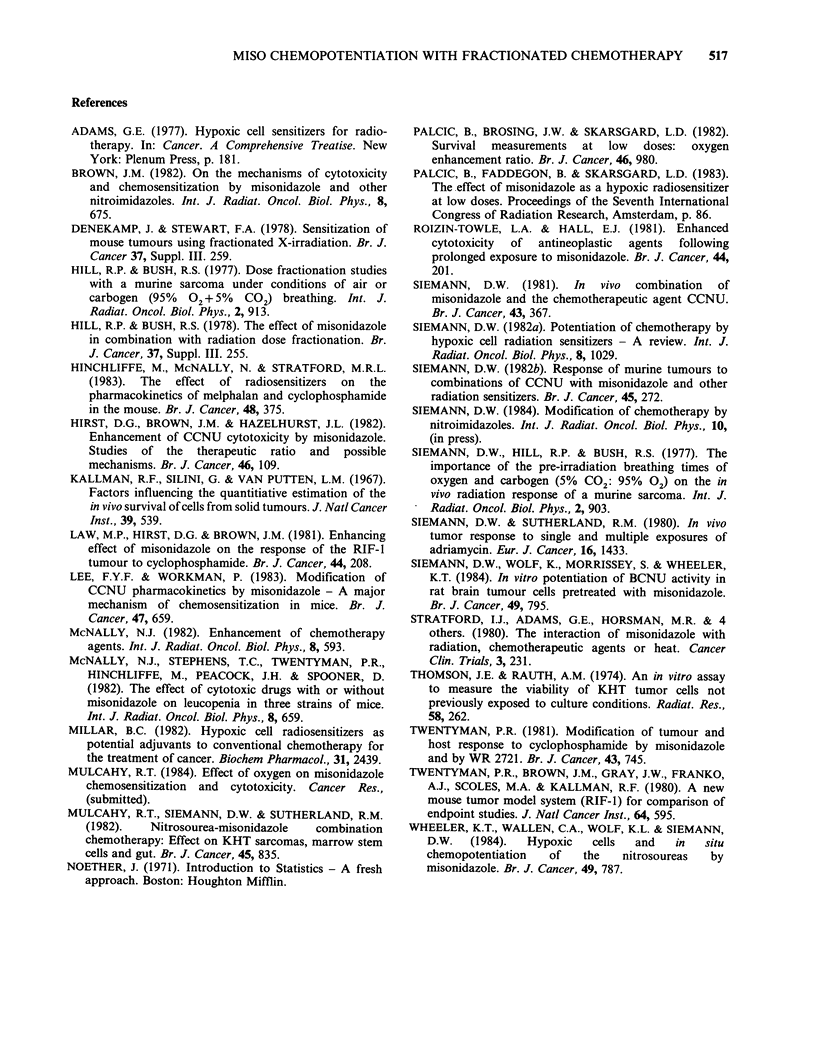# Chemopotentiation in vivo: no loss of sensitization with fractionation.

**DOI:** 10.1038/bjc.1984.208

**Published:** 1984-10

**Authors:** S. A. Hill, D. W. Siemann

## Abstract

The response of KHT sarcomas to one, two, five or ten daily fractions of 1-(2-chloroethyl)-3-cyclohexyl-1-nitrosourea (CCNU), with and without misonidazole (MISO), was evaluated using delay of tumour regrowth as the measure of response. When CCNU was given as 2 dose fractions separated by 24 h rather than as a single treatment, no extra dose was necessary to achieve a particular level of damage, suggesting a lack of damage repair. With increasing fraction number, however, an increasing total dose of drug was required to achieve a given effect, presumably to compensate for proliferation. Increasing drug doses also were readily tolerated (almost twice the LD50/7 for a single dose of CCNU resulted in no deaths when given in a 10 fraction treatment) indicating a large sparing of normal tissue toxicity when CCNU treatments were fractionated. The addition of MISO enhanced the tumour response to CCNU in all treatment schemes. When single doses of CCNU were combined with 0.5 mg g-1 MISO, an enhancement ratio (ER) of approximately 1.5 was observed. This ER was maintained for all fractionated treatment schedules including the 10 daily fraction protocol. In addition, no loss of sensitization with increasing fractionation was observed when a lower dose of 0.2 mg g-1 MISO was combined with each of 5 or 10 daily fractions of CCNU. Similar experiments were performed to test the combination of cyclophosphamide (Cy) and MISO (0.5 mg g-1) in the RIF-1 tumour; again chemopotentiation was maintained with increasing fractionation. These results of combined MISO and fractionated chemotherapy are in contrast to the rapid loss of sensitization observed when MISO is used as a radiation sensitizer and combined with small doses of X-rays, thus providing in vivo evidence of the mechanistic difference between the effects of MISO used as a radiation sensitizer or chemopotentiator. Peripheral white blood cell counts performed on mice receiving 5 daily fractions of CCNU +/- MISO displayed no significant enhancement of normal tissue toxicity by MISO. Thus combining MISO with repeated low dose treatments of a chemotherapeutic agent results in a therapeutic gain.


*Br. J. Cancer* (1984), **50**, 509–517

### Chemopotentiation *in vivo*: No loss of sensitization with fractionation

S.A. Hill & D.W. Siemann

*Experimental Therapeutics Division and Department of Radiation Oncology, University of Rochester Cancer Center, 601 Elmwood Avenue Box 704, Rochester, New York 14642, USA*

**Summary** The response of KHT sarcomas to one, two, five or ten daily fractions of 1-(2-chloroethyl)-3-cyclohexyl-1-nitrosourea (CCNU), with and without misonidazole (MISO), was evaluated using delay of tumour regrowth as the measure of response. When CCNU was given as 2 dose fractions separated by 24 h rather than as a single treatment, no extra dose was necessary to achieve a particular level of damage, suggesting a lack of damage repair. With increasing fraction number, however, an increasing total dose of drug was required to achieve a given effect, presumably to compensate for proliferation. Increasing drug doses also were readily tolerated (almost twice the LD50/7 for a single dose of CCNU resulted in no deaths when given in a 10 fraction treatment) indicating a large sparing of normal tissue toxicity when CCNU treatments were fractionated.

The addition of MISO enhanced the tumour response to CCNU in all treatment schemes. When single doses of CCNU were combined with $0.5\,mg\,g^{-1}$ MISO, an enhancement ratio (ER) of $\sim 1.5$ was observed. This ER was maintained for all fractionated treatment schedules including the 10 daily fraction protocol. In addition, no loss of sensitization with increasing fractionation was observed when a lower dose of $0.2\,mg\,g^{-1}$ MISO was combined with each of 5 or 10 daily fractions of CCNU. Similar experiments were performed to test the combination of cyclophosphamide (Cy) and MISO ($0.5\,mg\,g^{-1}$) in the RIF-1 tumour; again chemopotentiation was maintained with increasing fractionation. These results of combined MISO and fractionated chemotherapy are in contrast to the rapid loss of sensitization observed when MISO is used as a radiation sensitizer and combined with small doses of X-rays, thus providing *in vivo* evidence of the mechanistic difference between the effects of MISO used as a radiation sensitizer or chemopotentiator. Peripheral white blood cell counts performed on mice receiving 5 daily fractions of CCNU $\pm$ MISO displayed no significant enhancement of normal tissue toxicity by MISO. Thus combining MISO with repeated low dose treatments of a chemotherapeutic agent results in a therapeutic gain.

Over the last few years there has been increasing interest in potentiating the action of conventional chemotherapeutic drugs by the addition of a chemical radiosensitizing agent such as misonidazole (MISO). There are now many reports, using different combinations of these drugs and a variety of mouse tumour models, which indicate a greater tumour-cell cytotoxicity than that seen in the dose-limiting normal tissues i.e. a therapeutic gain has been achieved (for reviews see McNally, 1982; Siemann, 1982a).

As with the initial experimental evaluation of MISO as a radiation sensitizer, these chemo-potentiation studies have primarily concentrated on combinations of single doses of the chemo-therapeutic agent and sensitizer. While the single dose radiation studies demonstrated substantial enhancement ratios (Adams, 1977), when fractionated irradiations were investigated, the sensitizing effect of MISO decreased as the X-ray dose per fraction decreased (Denekamp & Stewart, 1978; Hill and Bush, 1978).

Currently little is known as to whether chemopotentiation by MISO is affected by drug treatment fractionation as has been observed for radiosensitization. This is of some clinical importance since, although chemotherapy treatment regimes differ widely according to the agent involved and the toxicities they produce, many involve multiple drug exposures. The present study therefore was undertaken to determine whether a loss of sensitization, similar to that seen with X-rays, also occurred when MISO was combined with fractionated chemotherapy. The combination of 1-(2-chlorethyl)-3-cyclohexyl-1-nitrosourea (CCNU) and MISO, was chosen as a model, for detailed investigation, since it is particularly effective in the KHT sarcoma (Siemann, 1981, 1982b). Studies designed to compare the effect of single doses and fractionated treatments (2, 5 and 10 daily fractions of CCNU in the absence or presence of MISO) on the KHT sarcoma were performed. These investigations therefore were directly comparable to previously reported sensitization studies combining fractionated radiotherapy and MISO treatment in this tumour model (Hill and Bush, 1978). For

Correspondence: S.A. Hill, Gray Laboratory, Mt. Vernon Hospital, Northwood, Middlesex, HA6 2RN, UK.
Received 9 May 1984; accepted 25 June 1984.

© The Macmillan Press Ltd., 1984

comparison, experiments were also performed to investigate the response of the RIF-1 tumour to multiple doses of cyclophosphamide (Cy) and MISO.

### Materials and methods

### *Mice and tumours*

The experiments described were performed using either the KHT sarcoma (Kallman *et al.,* 1967) or the RIF-1 tumour (Twentyman *et al.,* 1980) in female C3H/HeJ mice (8–14 weeks old), obtained from Jackson Laboratories, Bar Harbor, Maine. KHT cells were prepared from solid tumours by mechanical dissociation (Thomson & Rauth, 1974) and passaged *in vivo* every 2 weeks. RIF-1 tumour cells were maintained and passaged alternately *in vitro* and *in vivo* as described by Twentyman *et al.* (1980). For experiments, $2 \times 10^5$ cells were injected i.m. into the left hind legs of recipient mice. When the tumours reached 0.2–0.3 g in weight, the mice were randomly allocated to different treatment groups.

### *Drug treatments*

CCNU was dissolved in absolute ethanol (5 or $10\,mg\,ml^{-1}$), until just before injection, when it was diluted 10-fold by the addition of 9 ml of a 0.3% hydroxypropyl cellulose in sterile saline solution to 1 ml of the stock, solution. Cy was dissolved in saline at 2.5, 5 or $10\,mg\,ml^{-1}$. MISO was prepared as a $20\,mg\,ml^{-1}$ solution in sterile saline and given simultaneously with CCNU or Cy in the combined drug treatments. All injections were administered i.p. according to animal body wt.

### *Tumour response*

Following treatment, tumour response was assessed by measuring growth delay. Tumours were measured three to five times a week, by passing the tumour-bearing leg through a plastic rod with holes of increasing diameter. The smallest hole the tumour-bearing leg would pass through was recorded and converted to a tumour weight using a calibration curve (Siemann *et al.,* 1977; Siemann & Sutherland, 1980). The number of days for each tumour to grow to 5 times the size at treatment was then determined. The median time for the tumours of each group of animals to reach this endpoint was calculated and plotted against drug dose. Confidence limits about the median were calculated using non-parametric statistics (Noether, 1971).

### *Normal tissue toxicity*

Toxicity was assessed by measuring the depression of white blood cell (WBC) counts in both tumour- and non-tumour-bearing mice following treatment. Ten $\mu l$ blood samples were taken from the tips of the tails of unanaesthitized mice and diluted in 10 ml saline. Three drops of RBC lysing solution were added to lyse the red cells and counts were made on a Coulter Counter and Channelyzer system (Model C1000). Five mice were used for each treatment group.

Blood smears also were made, air-dried and stained with Wright's Giemsa stain. One hundred cells per slide then were counted and scored as granulocytes, lymphocytes or monocytes.

### *Pharmacokinetic determinations*

Ten minutes prior to blood collection, mice were injected with 0.2 ml of heparin. Blood was taken from the thoracic cavity after cutting the aorta and vena cava. Samples from 2–3 animals were collected and pooled. Following immediate high speed centrifugation, the plasma was withdrawn and frozen in liquid nitrogen.

For analysis 0.5 ml plasma was diluted with an equal volume of HPLC grade acetonitrile and spiked with a $10\,\mu l$ sample of an internal standard (Phenytoin, $2\,mg\,ml^{-1}$). After vortexing, centrifugation, and filtration, the supernatatant was injected into the high pressure liquid-chromatograph (Waters Associates, Milford, Mass). Reverse phase HPLC analysis was performed according to the methods of Lee and Workman (1983). Briefly, the separation of CCNU was achieved on a Waters Radical-PAK reverse phase bonded octadecylsilane (C18) cartridge column by running a two-step linear gradient starting at 34% acetonitrile in water changing to 44% acetonitrile in water over 5 min and to 64% acetonitrile in water over another 7 min as previously described (Lee and Workman, 1983). CCNU was analysed at a wavelength of 254 nm by means of a Waters Associates Model 441 u.v. absorbance detector.

### Results

Figure 1 shows the dose-response curves for the KHT sarcoma following treatment with one or two fractions of CCNU, either alone or in combination with MISO. These experiments were designed such that the total dose of both drugs remained constant, whether given as a single dose or as two equal fractions, separated by 24 h. A single curve can be drawn through the data for treatment with CCNU alone given as either one or two fractions. Similarly the data obtained when CCNU and MISO (a single dose of $1.0\,mg\,g^{-1}$ or two doses of $0.5\,mg\,g^{-1}$) were combined also fit a single curve. These results indicate that when CCNU was

**Figure 1** Median time to regrow to 5× the starting size as a function of CCNU dose for tumours treated with a single dose (○, ●) or two equal fractions (△, ▲) of either CCNU alone (○, △), or CCNU plus MISO (●, ▲). MISO was administered as a single dose of 1.0 mg g$^{-1}$ (●) or as two fractions of 0.5 mg g$^{-1}$ (▲). Each datum point represents the median tumour response (±98% confidence limits) on a group of 7 mice.

administered as two fractions over 24 h, the damage produced was no less than that seen when the tumours were treated with an equivalent single dose at time zero. In both instances the ratio of CCNU doses with and without MISO required to give the same regrowth time, i.e., the enhancement ratio (ER), was ~1.8. This value is similar to the ER of 1.9 previously reported for this tumour treated with single doses of CCNU and 1.0 mg g$^{-1}$ MISO (Siemann, 1982b).

To determine the effect of drug treatment fractionation on chemopotentiation by MISO, a fixed dose of MISO (0.5 mg g$^{-1}$) was combined with 1, 2, 5 or 10 daily doses of CCNU. Figure 2 shows the single dose and two fraction data. At a sensitizer dose of 0.5 mg g$^{-1}$ the enhancement of growth delay was a factor of ~1.5 for the single dose treatment and 1.7 for the two fraction treatment.

To investigate whether enhanced tumour responses could be maintained when daily 0.5 mg g$^{-1}$ doses of MISO were combined with even smaller doses of CCNU per fraction, five and ten fraction treatments were evaluated. In addition, for these latter protocols, the effect of reducing the MISO dose per fraction from 0.5 to 0.2 mg g$^{-1}$ also was studied. The results of these experiments are shown in Figure 3. A dose of 0.5 mg g$^{-1}$ MISO given with each CCNU dose resulted in enhancement ratios of 1.6 and 1.5 for the five and ten daily fraction protocols respectively (solid *vs* open symbols). When the lower dose of MISO (0.2 mg g$^{-1}$) was combined with daily doses of CCNU, enhanced tumour responses to CCNU also were observed (harlequin symbols). With this dose of MISO, both fractionation schedules led to ERs of 1.3–1.4. These values are similar to the previously published value of 1.3 measured for this tumour treated with a single dose of 0.25 mg g$^{-1}$ MISO plus CCNU (Siemann, 1982b). At both sensitizer doses studied the results therefore indicate no loss of sensitization with fractionation.

The various dose fractionation studies can be compared by determining an isoeffect curve. This is illustrated in Figure 4 where the total dose required for each CCNU fractionation schedule to yield a regrowth time of 20 days was calculated for both CCNU alone and CCNU combined with MISO. The data indicate that MISO potentiates the efficacy of CCNU not only at large doses per fraction but also that this potentiation is not lost as the dose per fraction is decreased.

This lack of a loss of potentiation with drug fractionation can also be seen in Figure 5. This figure illustrates the results obtained when a fixed total dose of 30 mg kg$^{-1}$ CCNU is delivered either as a single treatment or as two, five, six, eight or ten equal daily fractions, administered with or without 0.5 mg g$^{-1}$ MISO accompanying every dose. When 30 mg kg$^{-1}$ CCNU is subdivided into five fractions or more, the resulting growth delay approaches that measured for untreated control tumours i.e. daily doses of 6 mg kg$^{-1}$ CCNU or less are insufficient to retard tumour growth significantly. However, the addition of MISO to even these low doses of the nitrosourea, which in themselves are totally ineffective, produced a significantly enhanced tumour response.

In order to determine whether the observed potentiation of CCNU by MISO was influenced by changes in the pharmacokinetics of the chemotherapeutic agent in the presence of MISO, CCNU plasma levels were determined in C3H mice exposed to 20 mg kg$^{-1}$ CCNU alone or simultaneously combined with 0.2 or 0.5 mg g$^{-1}$ MISO (Table I). CCNU concentrations were

**Figure 2** Dose-response curves for tumours treated with one or two fractions of CCNU with (closed symbols), or without (open symbols) 0.5 mg g$^{-1}$ MISO. The two fraction data in the right-hand panel are reproduced from **Figure 1**. Data shown are the median tumour responses (±98% confidence limits) of groups of 7 mice. Upward arrows indicate groups in which animals had to be sacrificed before tumour regrowth.

**Figure 3** The response of tumours to five or ten daily fractions of CCNU alone (△, □) or CCNU plus 0.5 (▲, ■) or or 0.2 (▲, ■) mg g$^{-1}$ MISO, plotted as the median time to regrow to 5× their original weight (±98% confidence limits) for groups of 7 mice.

**Figure 4** Isoeffect curves for tumours treated with fractionated CCNU, administered either alone (○) or with 0.2 (◑) or 0.5 (●) mg g$^{-1}$ MISO accompanying each dose. The total dose of CCNU which resulted in tumour regrowth 20 days after treatment is plotted against fraction number.

**Figure 5** Median time to grow to 5× starting size (±98% confidence limits) *versus* fraction number, for groups of 7 mice treated with a total dose of $30\,mg\,kg^{-1}$ CCNU, with (●) or without (○) $0.5\,mg\,g^{-1}$ MISO accompanying each fraction.

**Table I** Effect of MISO on CCNU plasma pharmacokinetics[a]

| *CCNU* ($mg\,kg^{-1}$) | *MISO* ($mg\,g^{-1}$) | *CCNU plasma concentration at 25 min* ($\mu g\,ml^{-1}$)[b] |
|---|---|---|
| 20 | — | 0.15±0.02 |
| 20 | 0.2 | 0.15±0.02 |
| 20 | 0.5 | 0.24±0.03 |

[a]CCNU and MISO were administered simultaneously.

[b]Data shown are the mean ± s.e. of 6–7 determinations each pooling the blood of 2–3 mice.

determined 25 min after treatment using the procedure developed by Lee and Workman (1983). This time was chosen on the basis of previous evaluations (Lee and Workman, 1983) which demonstrated that monitoring the CCNU plasma concentrations at this fixed time could readily detect the influence of MISO on the plasma clearance of the nitrosourea. The data in Table I indicate that the plasma clearance of CCNU was reduced somewhat by a MISO dose of $0.5\,mg\,g^{-1}$ but not significantly affected by a dose of $0.2\,mg\,g^{-1}$.

To assess normal tissue toxicity, WBC counts were made on blood samples taken from the tails of C3H mice. Siemann (1982b) and McNally *et al.* (1982) have previously reported a depression in the white cell count which reached a nadir between days 2 and 4 following single dose treatment with CCNU or CCNU plus MISO. Since it was not known whether fractionation might influence the time course of this response, blood samples were taken at 2 or 3 day intervals, from groups of 5 animals, for a total of 26 days following the initial treatment. Measurements were made following the five fraction schedule only; each mouse received a total of $50\,mg\,kg^{-1}$ CCNU ($5\times10\,mg\,kg^{-1}$) with or without 0.2 or $0.5\,mg\,g^{-1}$ MISO accompanying each dose. All three treatment schedules produced a drop in the WBC count over the first 4 days, followed by a gradual recovery. However, none of the counts returned to pretreatment levels over the full three weeks of measurement. MISO did not significantly enhance the white cell depression measured at the nadir, although there was a suggestion that those animals which received the combined treatment maintained a somewhat lower WBC count than those animals receiving CCNU alone. No consistent difference was seen between the responses of tumour-bearing and non-tumour bearing mice.

Since white cell depression was maximal between days 4 and 7, a dose-response relationship for the five fraction regimen was measured on day 5, after the completion of treatment. Figure 6 shows total WBC counts for animals treated with a range of CCNU doses with or without 0.2 or $0.5\,mg\,g^{-1}$ MISO accompanying each dose. There was no consistent enhancement of CCNU-induced WBC toxicity by MISO.

Differential cell counts of granulocytes, lymphocytes and monocytes revealed that the cells primarily affected by the treatment where the peripheral granulocytes. Four days after the start of treatment, the depression in WBC count was almost entirely due to granulocyte depletion and no difference could be detected between samples from animals which had received CCNU alone or CCNU plus MISO.

Growth delay experiments also were performed to assess the response of the RIF-1 tumour to repeated daily doses of Cy with or without $0.5\,mg\,g^{-1}$ MISO. Complete dose response curves were obtained for protocols combining MISO with a range of 1, 2, 5 or 10 daily dose fractions of Cy. From these the ERs measured at a regrowth time of 15 days, were calculated (Table II). As was seen with CCNU, the degree of chemopotentiation measured remained unchanged whether MISO was combined with a single fraction or multiple daily dose fractions of Cy.

**Figure 6** Total white blood cell counts as a function of CCNU dose, measured after the final treatment in a five fraction regime. Groups of 5 mice were treated with varying doses of CCNU either alone (○) or with 0.2 (◑) or 0.5 (●) $mg g^{-1}$ MISO accompanying each dose.

**Table II** The enhancing effect of $0.5 mg g^{-1}$ MISO on the cytotoxicity of cyclophosphamide in the RIF-1 tumour, measured at a regrowth time of 15 days.

| *Treatment* | *Enhancement ratio* |
|---|---|
| Single dose | 1.4 |
| 2F | 1.4 |
| 5F | 1.5 |
| 10F | 1.3 |

### Discussion

That nitroimidazole radiation sensitizers can effectively increase the cytotoxicity of several chemotherapeutic agents is now well established (see McNally, 1982; Siemann, 1982a, 1984; Millar, 1982, for reviews). While most of the *in vivo* data concern tumour responses to large single doses of drugs, several studies have indicated that sensitization would not decrease with decreasing doses of the chemotherapeutic drug (Twentyman, 1981; Law *et al.*, 1981; Hirst *et al.*, 1982; Siemann, 1984). It also has been suggested that the therapeutic gain should be highest with low doses of chemotherapy, where MISO appears not to enhance bone marrow toxicity. Therefore, although clinical chemotherapy regimes are very varied and may or may not involve repeated drug doses, it was considered of interest to investigate whether both chemopotentiation and a therapeutic advantage could be maintained when treatments were extended to multiple drug exposures. The combinations of CCNU plus MISO and Cy plus MISO were chosen for evaluation because of their proven efficacy in single dose studies (for review see McNally, 1982).

The results presented here indicate that the potentiation of CCNU cell killing in the KHT sarcoma, achieved by a single administration of MISO, can be maintained at the same level with multiple fractions. This enhanced response was maintained with low doses per fraction of both agents. Significant sensitization was achieved with a MISO dose as low as $0.2 mg g^{-1}$ i.e. closer to clinically achievable levels than the doses generally employed in experimental investigations. A substantial delay in tumour regrowth also was seen when MISO was added to a low dose fractionated CCNU regime, which in itself produced no detectable antitumour activity (Figure 5).

The initial split-dose studies (Figure 1), showed that approximately equal levels of damage were produced by a particular drug dose whether it was delivered as a single treatment or as two equal fractions separated by 24 h, suggesting the absence of any repair during this interval. As treatments were extended to five and ten fractions (Figure 3) extra dose was required to produce the same tumour effect, but this was probably due to proliferation. The tumours continue to grow throughout the treatment period, since cell death and removal does not occur at the time each dose is administered. Due to the rapid growth of the KHT sarcoma, (volume doubling time 1.5–2.5 days), fractionation could not be extended beyond the 10 day treatment period employed. At many of the dose levels used in both the five and ten fraction experiments, no tumour regression could be detected by the end of the course of treatment, although ultimately shrinkage did occur, followed by regrowth. Clinically, lack of tumour response during treatment is frequently used as a reason for terminating therapy with the drug in question. Clearly in this experimental situation, lack of responsiveness during treatment did not indicate lack of treatment effectiveness and would have proved a poor prognostic indicator.

As well as the dose sparing effect observed in the tumour, a large sparing of lethal toxicity also was seen with increasing fractionation. Using mice of the same sex and strain as those in the present study, Siemann (1981), previously reported an LD50/7 value of 46.4 (44.4–48.6)(95% confidence limits) $mg\,kg^{-1}$ CCNU, which was reduced to 38.8 (36.9–40.8) $mg\,kg^{-1}$ on combination with $0.5\,mg\,g^{-1}$ MISO. In the present study greatly increased doses of drug were administered without approaching the LD50/7. No deaths occurred with a dose of $60\,mg\,kg^{-1}$ CCNU delivered in five fractions, or $90\,mg\,kg^{-1}$ in ten fractions i.e. practically double the dose which proved lethal to 50% of the animals when given as a single treatment. Similarly, when MISO was added to the treatment protocol, higher total doses of CCNU were tolerated when they were fractionated; 40 and $50\,mg\,kg^{-1}$ in five fractions and 50 and $80\,mg\,kg^{-1}$ in 10 fractions for 0.5 and $0.2\,mg\,g^{-1}$ MISO respectively. Since the doses did not extend to levels where any lethality was encountered, no estimate could be made of the increases in LD50 values resulting from the different protocols. However, the available data indicate that the increase in the tolerated dose is at least as large as the increase in the dose required to produce the same tumour effect. This suggests that there is no loss of therapeutic gain in progressing from one to ten fractions.

Myelosuppression is the primary dose-limiting toxicity of CCNU in man, which is manifest as a delayed leucopenia. Therefore, to further consider the question of therapeutic gain, we measured the total white cell count following treatment with CCNU with or without MISO, delivered in a five fraction protocol. The data illustrated in Figure 6, measured at the time of peak white cell depression, did not indicate any significant enhancement of CCNU-induced WBC toxicity by MISO, again suggesting no loss of therapeutic gain.

Two previously published studies have included information on the effects of five daily fractions of Cy and MISO. Twentyman (1981) considered just two dose levels of Cy and measured the growth delay resulting from treatment with and without MISO ($0.33\,mg\,g^{-1}$) in the RIF-1 and KHT sarcomas. Single dose experiments with this dose of MISO produced only a small enhancement of Cy response in both tumour systems. On extending the treatments to five fractions an increase in growth delay was measured at the higher of the two Cy doses for the RIF-1 tumour, but toxicity was very high at this drug level, even without the addition of MISO. However, the response of the KHT tumour was significantly enhanced at both Cy doses. Extrapolation of the data suggested an ER of 1.5 which was significantly greater than the enhancement achieved by a single treatment. In a similar experiment, again using the RIF-1 tumour, Law *et al.* (1981) reported an ER of 1.5 to 1.7 for the five fraction treatment with $0.3\,mg\,g^{-1}$ MISO. No data were available for single dose treatments with this level of MISO, and no measurement of normal tissue toxicity was made. These data together with the current results which are summarized in Table II, suggest that MISO can markedly increase the effectiveness of low as well as high dose Cy. Mulcahy *et al.* (1982) investigated the effectiveness of combining $0.7\,mg\,g^{-1}$ MISO with each of three daily doses of BCNU ($10\,mg\,kg^{-1}\,day^{-1}$) but no enhancement of growth delay was measured. Clearly more data are necessary to allow a thorough evaluation of which drug combinations are most likely to result in a favorable therapeutic response when delivered in a multiple low dose treatment regime.

The results presented here for the potentiation of CCNU damage by MISO are in direct contrast to the data obtained when MISO was tested as a radiation sensitizer (Hill & Bush, 1978; Denekamp & Stewart, 1978). In these studies, large enhancement ratios were measured for single dose treatments, however, when the radiation dose was fractionated, the sensitizing effect of the drug decreased as the radiation dose per fraction decreased. Similarly, breathing carbogen (95% $O_2$ + 5% $CO_2$) was found to sensitize tumours to the effects of large single doses of radiation (by decreasing the hypoxic fraction), but again, sensitization decreased with fractionation and decreasing doses of radiation (Hill & Bush, 1977). The current data emphasize that the mechanism by which MISO potentiates the action of CCNU must be different from that by which both it and oxygen act as radiation sensitizers.

Several reasons have been put forward to explain the loss of radiation sensitization with decreasing dose per fraction. At low doses of radiation, the oxygen enhancement ratio (OER) has been found to be reduced (Palcic *et al.*, 1982). This would tend to imply that multiple small fractions should be more effective than large single doses of radiation, since the protected status of the hypoxic cells would be reduced, this would also reduce the degree of radiosensitization which be achieved. In addition, since it is the fully oxygenated radiosensitive cells within a tumour which will dominate its response when low radiation doses are used, the natural process of reoxygenation occuring between successive fractions may also serve to reduce the importance of hypoxic cells and therefore the benefit that might be gained by hypoxic cell sensitizers such as MISO. This is further supported by the observation that MISO affects the final, but not the initial slope of a radiation survival curve i.e. the $\beta$ but not the the $\alpha$ component of the linear quadratic cell survival model (Palcic *et al.*, 1983). Since the $\alpha$ term dominates at low radiation doses, these data would again imply that MISO should be more effective at high radiation doses.

Several studies have suggested that MISO is a more efficient chemosensitizer when combined with low doses of the chemotherapeutic agent (Twentyman, 1981; Law *et al.*, 1981; Hirst *et al.*, 1982; Mulcahy *et al.*, 1982). It also has been noted, both *in vitro* and *in vivo,* that chemopotentiation frequently, though not always, occurs as a reduction or loss of the shoulder on the dose response curve, with little change in the final slope (Stratford *et al.*, 1980; Siemann *et al.*, 1984; Roizin-Towle & Hall, 1981). This suggested that fractionation might prove successful. The data presented here have indicated a cumulative effect of repeated low doses, with no evidence of repair processes occurring between subsequent fractions. Potentiation of CCNU damage appears to occur with every treatment (Figure 4), even when the chemotherapy alone is totally ineffective in retarding tumour growth (Figure 5). One explanation may be that chemosensitization occurs at least to some extent at oxygen levels above those required for radiobiological hypoxia. If this were the case a large population of cells would be at risk to be sensitized and hence even at the lowest CCNU dose per fraction evaluated this subpopulation could still determine the overall tumour response, therefore no loss of chemopotentiation with fractionation would be expected. In support of this view, *in vitro* studies have indicated that chemopotentiation, although reduced in magnitude, can be demonstrated at relatively high oxygen tensions despite the $K_M$ value for chemopotentiation by MISO being equal to that for MISO cytotoxicity (Mulcahy, 1984). In addition it seems clear that, while hypoxic cells are necessary for chemopotentiation (McNally, 1982; Brown, 1982; Wheeler *et al.*, 1984; Siemann, 1984) this effect is not restricted to the fraction of radiobiologically hypoxic tumour cells (Siemann, 1982a). Finally, if modification of chemotherapeutic agent activity occurred at oxygen tensions above those associated with radiobiological hypoxia this also could play a role in the enhanced normal tissue toxicities associated with drug-sensitizer combinations.

Possible mechanisms for chemopotentiation have been extensively reviewed elsewhere (Brown, 1982; Millar, 1982; McNally, 1982; Siemann, 1982a, 1984) and will not be addressed in detail here. It is possible however, to consider the importance of modification of cytotoxic drug pharmacokinetics by MISO in the context of the present experiments. Considerable data now exist to indicate that sensitizers can alter the pharmacokinetics of a number of chemotherapeutic agents (Hinchliffe *et al.*, 1983; Lee & Workman, 1983). While failing to account for all the chemopotentiation results, pharmacokinetic changes are involved when chemotherapeutic agent efficacies are enhanced *in vivo* by large sensitizer doses (for review see Siemann, 1984). Lee and Workman (1983) observed that the plasma clearance of CCNU was reduced as the MISO dose was increased, with a threshold MISO dose of approximately $0.3\,mg\,g^{-1}$. The current results (Table I). which indicate that a MISO dose of $0.5\,mg\,g^{-1}$ reduced the clearance of CCNU while a $0.2\,mg\,g^{-1}$ dose had no effect, are consistent with those previous findings. The $0.2\,mg\,g^{-1}$ dose was sufficient, however, to achieve chemopotentiation in a fractionated dose protocol (Figures 3 and 4) suggesting that chemopotentiation can occur in the absence of a pharmacokinetic effect.

In summary, the present results indicate that unlike the loss of sensitization seen when MISO is added to fractionated radiation, no loss of potentiation occurred with fractionated CCNU plus MISO. In addition, repeated low dose treatments with both agents did not appear to increase the normal tissue toxicity above that achieved with a single high dose treatment, suggesting no loss of therapeutic benefit with fractionation. Further fractionated treatments aimed at determining the effectiveness of other drug combinations particularly with clinically relevant doses of MISO and utilizing several tumour models are indicated. Nevertheless, the benefits seen in this study suggest that MISO might be successfully added to repeated low dose chemotherapy treatments.

The authors thank Dr R.T. Mulcahy for helpful discussions and criticisms of this work. CCNU was kindly provided by Dr Robert Engle of the Developmental Therapeutics Program, Division of Cancer Treatment of the National Cancer Institute. MISO was obtained from Dr Ven Narayanan of the Drug Synthesis and Chemistry Branch, National Cancer Institute. We also thank Barbara Granger for the preparation of the manuscript. These investigations were supported by NIH grants CA-11051, CA-20329 and CA-11198.

### References

ADAMS, G.E. (1977). Hypoxic cell sensitizers for radiotherapy. In: *Cancer. A Comprehensive Treatise*. New York: Plenum Press, p. 181.

BROWN, J.M. (1982). On the mechanisms of cytotoxicity and chemosensitization by misonidazole and other nitroimidazoles. *Int. J. Radiat. Oncol. Biol. Phys.*, **8**, 675.

DENEKAMP, J. & STEWART, F.A. (1978). Sensitization of mouse tumours using fractionated X-irradiation. *Br. J. Cancer* **37**, Suppl. III. 259.

HILL, R.P. & BUSH, R.S. (1977). Dose fractionation studies with a murine sarcoma under conditions of air or carbogen (95% $O_2$+5% $CO_2$) breathing. *Int. J. Radiat. Oncol. Biol. Phys.*, **2**, 913.

HILL, R.P. & BUSH, R.S. (1978). The effect of misonidazole in combination with radiation dose fractionation. *Br. J. Cancer*, **37**, Suppl. III. 255.

HINCHLIFFE, M., MCNALLY, N. & STRATFORD, M.R.L. (1983). The effect of radiosensitizers on the pharmacokinetics of melphalan and cyclophosphamide in the mouse. *Br. J. Cancer*, **48**, 375.

HIRST, D.G., BROWN, J.M. & HAZELHURST, J.L. (1982). Enhancement of CCNU cytotoxicity by misonidazole. Studies of the therapeutic ratio and possible mechanisms. *Br. J. Cancer*, **46**, 109.

KALLMAN, R.F., SILINI, G. & VAN PUTTEN, L.M. (1967). Factors influencing the quantitiative estimation of the *in vivo* survival of cells from solid tumours. *J. Natl Cancer Inst.*, **39**, 539.

LAW, M.P., HIRST, D.G. & BROWN, J.M. (1981). Enhancing effect of misonidazole on the response of the RIF-1 tumour to cyclophosphamide. *Br. J. Cancer*, **44**, 208.

LEE, F.Y.F. & WORKMAN, P. (1983). Modification of CCNU pharmacokinetics by misonidazole – A major mechanism of chemosensitization in mice. *Br. J. Cancer*, **47**, 659.

MCNALLY, N.J. (1982). Enhancement of chemotherapy agents. *Int. J. Radiat. Oncol. Biol. Phys.*, **8**, 593.

MCNALLY, N.J., STEPHENS, T.C., TWENTYMAN, P.R., HINCHLIFFE, M., PEACOCK, J.H. & SPOONER, D. (1982). The effect of cytotoxic drugs with or without misonidazole on leucopenia in three strains of mice. *Int. J. Radiat. Oncol. Biol. Phys.*, **8**, 659.

MILLAR, B.C. (1982). Hypoxic cell radiosensitizers as potential adjuvants to conventional chemotherapy for the treatment of cancer. *Biochem Pharmacol.*, **31**, 2439.

MULCAHY, R.T. (1984). Effect of oxygen on misonidazole chemosensitization and cytotoxicity. *Cancer Res.*, (submitted).

MULCAHY, R.T., SIEMANN, D.W. & SUTHERLAND, R.M. (1982). Nitrosourea-misonidazole combination chemotherapy: Effect on KHT sarcomas, marrow stem cells and gut. *Br. J. Cancer*, **45**, 835.

NOETHER, J. (1971). Introduction to Statistics – A fresh approach. Boston: Houghton Mifflin.

PALCIC, B., BROSING, J.W. & SKARSGARD, L.D. (1982). Survival measurements at low doses: oxygen enhancement ratio. *Br. J. Cancer*, **46**, 980.

PALCIC, B., FADDEGON, B. & SKARSGARD, L.D. (1983). The effect of misonidazole as a hypoxic radiosensitizer at low doses. Proceedings of the Seventh International Congress of Radiation Research, Amsterdam, p. 86.

ROIZIN-TOWLE, L.A. & HALL, E.J. (1981). Enhanced cytotoxicity of antineoplastic agents following prolonged exposure to misonidazole. *Br. J. Cancer*, **44**, 201.

SIEMANN, D.W. (1981). *In vivo* combination of misonidazole and the chemotherapeutic agent CCNU. *Br. J. Cancer*, **43**, 367.

SIEMANN, D.W. (1982*a*). Potentiation of chemotherapy by hypoxic cell radiation sensitizers – A review. *Int. J. Radiat. Oncol. Biol. Phys.*, **8**, 1029.

SIEMANN, D.W. (1982*b*). Response of murine tumours to combinations of CCNU with misonidazole and other radiation sensitizers. *Br. J. Cancer*, **45**, 272.

SIEMANN, D.W. (1984). Modification of chemotherapy by nitroimidazoles. *Int. J. Radiat. Oncol. Biol. Phys.*, **10**, (in press).

SIEMANN, D.W., HILL, R.P. & BUSH, R.S. (1977). The importance of the pre-irradiation breathing times of oxygen and carbogen (5% $CO_2$: 95% $O_2$) on the *in vivo* radiation response of a murine sarcoma. *Int. J. Radiat. Oncol. Biol. Phys.*, **2**, 903.

SIEMANN, D.W. & SUTHERLAND, R.M. (1980). *In vivo* tumor response to single and multiple exposures of adriamycin. *Eur. J. Cancer*, **16**, 1433.

SIEMANN, D.W., WOLF, K., MORRISSEY, S. & WHEELER, K.T. (1984). *In vitro* potentiation of BCNU activity in rat brain tumour cells pretreated with misonidazole. *Br. J. Cancer*, **49**, 795.

STRATFORD, I.J., ADAMS, G.E., HORSMAN, M.R. & 4 others. (1980). The interaction of misonidazole with radiation, chemotherapeutic agents or heat. *Cancer Clin. Trials*, **3**, 231.

THOMSON, J.E. & RAUTH, A.M. (1974). An *in vitro* assay to measure the viability of KHT tumor cells not previously exposed to culture conditions. *Radiat. Res.*, **58**, 262.

TWENTYMAN, P.R. (1981). Modification of tumour and host response to cyclophosphamide by misonidazole and by WR 2721. *Br. J. Cancer*, **43**, 745.

TWENTYMAN, P.R., BROWN, J.M., GRAY, J.W., FRANKO, A.J., SCOLES, M.A. & KALLMAN, R.F. (1980). A new mouse tumor model system (RIF-1) for comparison of endpoint studies. *J. Natl Cancer Inst.*, **64**, 595.

WHEELER, K.T., WALLEN, C.A., WOLF, K.L. & SIEMANN, D.W. (1984). Hypoxic cells and *in situ* chemopotentiation of the nitrosoureas by misonidazole. *Br. J. Cancer*, **49**, 787.